# Memoirs of the Royal Academy of Medicine of Paris

**Published:** 1846-04

**Authors:** 


### Art. VII.

*Mémoires de l'Académie Royale de Médecine.* Tome XI.—*Paris,* 1845.

*Memoirs of the Royal Academy of Medicine of Paris.* Vol. XI.—*Paris,* 1845. 4to, pp. 684.

Of the three *éloges* with which the present volume of Memoirs opens, there is one which will be read with deep interest,—we mean that of which Esquirol forms the theme. Strong and enthusiastic as are the epithets which flow from the facile pen of M. Pariset, they do not give an exaggerated picture of the talents and the virtues of the man;—the simple events of his life, the respect with which his memory is honoured in his native land, the impulse he gave to the philosophical study of the terrible maladies with which his name is indissolubly connected, and the vast improvements he introduced in the actual practical comprehension of those maladies, his tried benevolence, charity and lovingness of character, all give warranty to the encomiums of that accomplished writer. It is good, it is gladdening to read all this,—and to know that eminence, distinction, and respect, such as Esquirol gained, may be achieved by a man who started originally in life with scarcely a sou in his pocket.

Of M. Double and M. Bourdois de la Motte, whose lives are likewise briefly narrated, the latter was very little known beyond his own immediate sphere; the former, too, had long ceased to mingle much in medical circles, though a member both of the Institute and Academy of Medicine.

Although we retain unchanged the feeling which sometime since led us to decry the system of indiscriminate laudation adopted in France, in regard of defunct members of learned societies, still, we believe, that it is both wise and proper that some record should be kept of the lives of all the deceased members of our profession. For this reason, we perceive with considerable satisfaction, that the editor of the 'London Medical Directory,' has this year commenced the plan of briefly recording the ascertainable particulars of the lives of those whom the preceding twelve months may have consigned to the grave.

M. Frederic Dubois supplies an interesting essay on the recent progress of medicine and of surgery in France; this will be read with advantage, but does not bear condensation.

I. *Memoir on urethroplasty,* by M. Ségalas. Under this title M. Ségalas describes the modifications which he has introduced in the mode of applying plastic surgery to cure of openings in the urethra. In a letter, which this surgeon addressed to M. Dieffenbach in 1840, he endeavoured to show that the frequent failure of urethro-plastic operations depended upon the great difficulty experienced in preventing the contact of the urine with the parts which the operator proposes to unite; and that hence the first condition of success was the establishment of a temporary channel for the discharge of the urine through the perineum, by means of a gum-elastic bougie retained in the wound. In the present publication, he more particularly seeks to give additional support to his views by the narration of another (his second) successful case.

To detail all the circumstances of the operation would occupy more space than we can afford; but a few particulars will suffice to make the general nature of M. Ségalas' proceeding comprehensible. The patient, a working shoemaker, aged about 30, had lost a considerable portion of the spongy part of the urethra at the age of six years, the missing part having sloughed away in consequence of the owner having playfully tied up the penis with a string. The entire thickness of the urethra, for about an inch in length, was wanting, when the operation was undertaken; the urine and semen of course passed by the posterior opening. The portion of urethra lying in front of the hiatus described, was contracted, but perfectly pervious. The man's general heath was excellent.

The operator commenced (21st July 1841) by placing a small gum-elastic bougie in the urethra. Finding that this instrument caused no particular inconvenience to the patient, though allowed to remain *in situ* for a few days, M. Ségalas, at the expiration of these, incised the prepuce at its upper part, in order to give all possible freedom to the structures to be brought in contact,—a preliminary step, the more important as the patient was affected with phymosis. Six days after this little operation (which produced not the slightest constitutional disturbance), M. Ségalas opened the membranous part of the urethra through the perineum, passed a gum-elastic sound through into the bladder, and then by means of an œsophagus-tube, transforming this sound into a siphon, caused the urine to pass away continuously through the perineum. Sixteen days after (the wound in the latter place having ceased to give the least pain, the patient being in good health, and not annoyed by erections), the closing step of this complicated operation was performed as follows:

"I made two semi-elliptical incisions in a transverse direction on the inferior surface of the penis, in front of and below the hiatus caused by the loss of substance: these incisions met each other on each side of the lateral aspect of the corpus cavernosum. I then carefully denuded the entire surface circumscribed by these two incisions, and bringing together the two lips of the wound thus formed, I united them by means of six points of twisted suture, one on the median line, three to the right, and two to the left. My intention had been to make a third point of suture on the latter side; but an observation made by one of the persons present, that the swelling might cause too great an amount of compression between the parts united, prevented me from doing this, and I left a much wider space between the two needles in question, than between those of the opposite side"

M. Ségalas attaches much importance to this omission, tracing to it the difficulty subsequently encountered in closing a small fistulous opening in the site, where he had proposed, but omitted, to place an additional needle. A minute opening still existed in the month of February following, which was finally closed after repeated failures by cauterization, by a point of twisted suture. The cure continued perfect, when the patient was last seen in July 1844. Three engravings exhibit the progress of events in connexion with the operation. Nothing is said of the effects of the cicatrices on erection of the penis,—a matter of no small importance.

II. *An Essay on the two diseases known under the name of meningeal apoplexy*, by Dr. R. Prus. These two diseases are hemorrhage between the arachnoid and pia mater: and hemorrhage into the cavity of the serous membrane. Both varieties are illustrated by a certain number of cases (not remarkable for closeness of description in any respect); and the relation of these is followed by a general account of the affections.

A. To commence with the first, whether *blood effused into the subarachnoid tissue* has made its way there in consequence of exhalation or rupture of a vessel, it may be found in clots or liquid, may be spread in a layer on the outer surface of the convolutions or penetrate into the anfractuosities, and even pass through the substance of the pia mater. The most extensive effusions occur at the base of the skull, and are most frequently due to rupture of an artery. A peculiarity in these effusions is that, although the progress of the symptoms render it unquestionable, that escape of blood may have, and sometimes must have, taken place on two or more successive occasions, no differences exist in different parts of the extravasated blood significant of a difference of age: the solution of the difficulty suggested by the author, namely, that the want of such appearance depends on the short duration of the disease, is not altogether satisfactory, as indeed he himself admits. The formation of pseudo-membrane round the effused blood is slow to occur in this situation: a case related by Morgagni, shows that no trace of its development may be discoverable even eight days after the hemorrhage has occurred. The author conjectures that the deficiency of false membrane may depend on the flux and reflux of the cephalo-rachidian fluid; but to the movement of this fluid, he conceives, the disappearance of blood effused in this situation (when not in sufficient quantity to cause death) is possibly altogether due. Coagula contained within the ventricles become invested with pseudo-membrane.

Hemiplegia is a very rare effect of the description of hemorrhage under consideration. Of twelve cases of rupture of arteries, two only were attended with this symptom; and in no instance was rupture of the veins or sinuses productive of paralysis. This peculiarity may very probably depend, as M. Prus observes, either, 1st, upon the fact that the sub-arachnoid tissue being naturally destined to receive the cephalo-rachidian fluid, is capable of giving lodgement to an undue quantity of blood without ill effects; or 2dly, that the effused blood being spread over a large surface by that fluid, exercises no special pressure upon any point in particular of the cerebral surface. In a certain class of cases, however, where a very large quantity of blood is thrown out, such marked compression of the brain arises, that sudden and complete annihilation of power generally takes place, and in consequence no local paralysis can be particularly traced.

In the cases observed by M. Prus the only invariable symptom was coma, preceded several times by general discomfort and prostration. Cephalalgia, and redness and heat of the integuments of the face, are rather to be considered in the light of premonitory symptoms, than evidences of the actual occurrence of hemorrhage. Slight delirium was noticed in one case; the intellectual faculties are scarcely ever perverted, but for the most part weakened.

The course of the disease, commonly continuous, is in some instances intermittent,—obviously a consequence of successive hemorrhages taking place. Death occurs more rapidly in certain cases of sub-arachnoid hemorrhage than of any form of effusion of blood into the cerebral structure itself; the prognosis, too, seems absolutely without hope, as death was the invariable result, either immediately or within the space of eight days, in the cases brought together by the author. No appearance of cysts has yet been discovered in the sub-arachnoid cavity.

B. *Effusion of blood into the cavity of the arachnoid,* always occurs by exhalation, and not in consequence of rupture of vessels. The blood does not stain the cephalo-rachidian fluid,—seeing that it does not come into contact with it; and, when it has been extravasated some five days or so, is invariably invested with a pseudo-membrane. Differences in the characters of different parts of the coagula, significant of differences in the periods of effusion of the blood, have frequently been noticed. Intra-arachnoid coagula, generally adhere closely and firmly to the external lamina of the serous membrane; traces of absorption through the agency of cysts have sometimes been discovered.

It will be observed, that all these anatomical conditions are different from those noticed in sub-arachnoid hemorrhages; and a corresponding difference in symptoms may be traced. Paralysis, at least of movement, is common, having occurred in six of eight cases related by M. Prus; paralysis of sensation is less usual. In three of these eight cases sudden loss of consciousness occurred. Somnolence and coma occur almost invariably towards the close of cases of both these forms of hemorrhage; but in the intra-arachnoid form, cephalalgia, dryness of the tongue, fever, and delirium, are almost always observed,—and these symptoms are doubtless the effects of inflammation of the arachnoid, itself due to congestion either preceding the hemorrhage or caused by the irritative action of the effused blood. Delirium commonly occurs about the fourth day in cases of intra-arachnoid hemorrhage,—the precise period at which the investiture of the clot with pseudo-membrane becomes apparent.

M. Prus contrasts the symptomatic characters of meningeal, with those of cerebral hemorrhage, somewhat in the following wise. Sudden loss of consciousness existed in three (or rather but two only, as in one of the three death was immediate) out of fourteen cases of meningeal apoplexy; it is unnecessary to state how different the proportion of cases would be in cerebral hemorrhage.

The paralysis of movement occurring in intra-arachnoid hemorrhage, is not generally as complete as in effusion into the cerebral substance,—and is but rarely, and then temporarily only, accompanied with paralysis of sensation. Again, paralysis of movement, resulting from meningeal apoplexy, may completely disappear,—a most rare result in old subjects, where the symptom is produced by cerebral hemorrhage. Deviation of the mouth, so common in the latter species of effusion, is extremely rarely met with in cases of meningeal apoplexy; in the single case in which M. Prus observed the symptom, the patient had previously had hemorrhage of the brain itself. In fine, somnolence and coma constitute, from their intensity and continuance, one of the principal characters of meningeal apoplexy.

The duration of the sub-arachnoid apoplexy does not, according to the experience of M. Prus, exceed eight days; it is always fatal. The duration of intra-arachnoid hemorrhage may extend to a month and upwards; and recovery may take place, as shown by the discovery of cysts in the cavity of the serous sac.

The only point regarding treatment to which M. Prus' observations would lead, is that in cases of intra-arachnoid hemorrhage, active and sustained antiphlogistic measures should be put in force, for the purpose of controlling the arachnitis commonly ensuing. Although this result is sufficiently sterile, the fault rests not with M. Prus; some totally new light must be thrown upon the physiology and pathology of the cerebral organs, before any solid improvement in the treatment of the affections to which they are subject can fairly be expected.

The essay we have analysed is a useful production;—it certainly contains no single fact or inference which might not have been proved with the aid of cases collected by persons who observed long before M. Prus; but it has brought together and placed in a fit condition for comparison various facts which had previously not been so strikingly arranged beside each other. The author is unjustly silent concerning the observations of MM. Cruveilhier and Durand-Fardel; and omits all mention of Dr. Carswell's clear description of intra-arachnoid hemorrhage, probably from the pressure of that species of literary ignorance, which, originating partly in vanity, partly in dishonesty, has become characteristic of the French school of medical literature.

III. *Essay on œdema of the glottis,* by M. Valleix. The essay of M. Valleix was composed in answer to a question put by the Academy of Medicine of Paris, to the following effect: What are the causes of œdematous laryngeal angina (œdema of the glottis); its mode of progress, successive symptoms and "differential diagnosis;" what is the fitting treatment of the disease, and what the advantages and the disadvantages of tracheotomy?

Forty-three cases, collected from different sources, form the basis of the direct description of the disease given by M. Valleix; and he has availed himself of nine other cases in examining the subject of diagnosis. A short inquiry into the literature of the subject, and into the claims of Boerhaave and Bayle as the first describers of the disease, precedes the account of its anatomy: Bayle is pronounced the original historian of the disease.

M. Valleix well observes that, with whatever justness the charge may frequently be urged against the pursuits of morbid anatomy, that the detection of anatomical changes explains but little of the nature and phenomena of diseases, such a charge is totally pointless in the case of œdema of the glottis. The anatomical changes here, in truth, constitute the whole malady: take away these, and the total disease is gone. Hence the importance of closely and thoroughly examining them.

The infiltration of the sub-mucous tissue of the glottis may be simply and purely serous, sero-purulent, or actually purulent—a fact showing the necessity of giving a somewhat different title to the disease than that in habitual use. Pure serous infiltration is comparatively rare; some cases of the kind are recorded in which the laryngeal œdema arose in the course of general anasarca. Now, in these cases, the persons who observed them were of opinion that the laryngeal œdema was a consequence of the general state, and had occurred quite independently of local inflammation. A case, transcribed from a weekly print by M. Valleix, contains a strong asseveration on the part of at least four competent persons, that not the smallest trace of inflammation could be detected in the part. But the author, not content with this statement, proceeds to show, as he conceives, that inflammatory action might have been present, and left no anatomical evidence of its existence. We confess ourselves unable to descry the utility or philosophy of thus contending against the admission of plain and obvious facts.

In more than three fourths of the cases brought together by this author, the infiltrated fluid was either purulent or sero-purulent. In such cases the upper opening of the larynx is more or less completely plugged with a soft circular fold of tumid membrane, somewhat larger generally on one side than the other. The epiglottis is pushed upwards towards the base of the tongue, and its edges sometimes included in the infiltration. The mucous membrane may be perfectly pale.

With regard to the localization of the infiltrated fluid, M. Valleix finds that, in twenty-four of thirty-two cases the aryteno-epiglottic ligaments were distinctly affected; in the other eight cases no very accurate limitation of the affected parts is made, but in all the edges, the *lips* or the *periphery* of the glottis are said to have been implicated. In four individuals the infiltration had reached the epiglottis; in one, the uvula; in two, the pharynx generally. The abundance of fluid greatly varies; nor is it possible to supply any very just measure of its amount as a general guide. M. Louis has observed some cases which would appear to show that a certain quantity of fluid may disappear from this site either after death or during the closing moments of existence; at least, he has found the aryteno-epiglottic ligaments *wrinkled* in such manner, as to make it probable that they had previously been distended by fluid. At the opening of the glottis are seen two swellings, more or less rounded and prominent, and tending, where of large size, to fall downwards on the upper opening of the larynx. A remarkable fact concerning these swellings, and their tendency to fall downwards, is, that the air is readily enabled to escape outwards by pushing them aside, whereas, the more strong the effort to force air inwards, the more effectual becomes the morbid resistance to its passage. Experiments on the larynx after death in these cases have demonstrated these facts in the clearest manner, and their obvious connexion with some of the peculiar symptoms of the disease cannot be overlooked.

The mucous membrane of the larynx was healthy in *three* cases only, in the parts corresponding to the seat of the serous infiltration; in two of these cases, however, the cartilages were diseased. In *six* other cases the mucous membrane is simply said to have been discoloured; but in five of the six there existed either a morbid state of the cartilages, ulceration of the trachea, or inflammation of some part adjoining the glottis. In *all the remaining cases* the mucous membrane exhibited distinct anatomical alteration. It was red in one third of the subjects, ulcerated in about one sixth of them, and raised up, dissected, as it were, from the subjacent parts by pus. M. Valleix has in one instance seen it greatly softened. In one case greenish discoloration existed; in another three obvious patches of gangrenous character.

Next to the mucous membrane the cartilages were most frequently affected, suffering in sixteen cases, or upwards of one half of the whole. Six times they were carious, or even, in one case, destroyed to a certain extent. The cricoid cartilage is that most frequently affected. The various changes observed appertain to laryngeal phthisis, which is here, M. Valleix observes, a powerful cause of œdema of the glottis; but it may be observed that M. Louis met with but three examples of œdema of the glottis among all his post-mortem examinations of consumptive patients.

Passing over some other anatomical changes, less important on account of their rarity than those noticed, we may state, as the result of the analysis of facts undertaken by M. Valleix, that there are but a very small number of cases indeed, in which it can, even on first view, be supposed that œdema of the glottis had occurred independently of organic change, and that in these very few cases, close examination of the entire history gives motive for doubting the real absence of such change. Hence it would follow that the various morbid states enumerated are the real immediate causes of the infiltration constituting œdema of the glottis.

The ages of 36 patients were as follows:

| Ætat. | Cases. | Ætat. | Cases. |
|---|---|---|---|
| 0 to 10 | 2 | 40 to 50 | 8 |
| 10 20 | 5 | 50 60 | 5 |
| 20 30 | 8 | 60 70 | 3 |
| 30 40 | 4 | 71 | 1 |

The five cases set opposite the decennial period, ten to twenty, occurred in persons aged from 18 to 20 years. It is rendered probable by these facts, that from the eighteenth to the thirtieth years is the period at which the tendency to this disease reaches its maximum. Males and females suffer with different degrees of frequency; the former furnished 29 cases, the latter 11. The facts collected by the author, bearing upon constitution, temperament, and season of the year, as causes of the disease, are, as well from their small number as from other causes, completely without signification. This he admits.

The considerations into which the author enters on the subject of the state of health existing at the period of occurrence of the œdema are of practical interest. In four of forty cases the individuals were in the enjoyment of good health when the disease appeared; in other words, in one tenth of cases of its occurrence it is a phenomenon or effect of simple angina. In but one of these did the attack prove fatal. A case of the kind is related, in which life was saved by tracheotomy, timeously performed.

The cases of non-laryngeal disease, in which the œdema supervenes, were as follows:

| | |
|---|---|
| During the course of or convalescence from typhoid fever . . . | 10 |
| During convalescence from pneumonia . . . . . . | 4 |
| In erysipelas . . . . . . . . . | 1 |
| After scarlatina . . . . . . . . . | 1 |
| After lithotomy . . . . . . . . . | 1 |
| During the treatment of a fracture, accompanied with fever . . . | 1 |
| During convalescence from cerebral congestion . . . . . | 1 |
| During an attack of pulmonary catarrh . . . . . . | 1 |
| In hypertrophy of the heart . . . . . . . | 1 |
| In elephantiasis . . . . . . . . . | 1 |

The diseases particularly exercising their action on the larynx, or actually primarily affecting that organ, were these:

| | |
|---|---|
| Laryngeal phthisis, without pulmonary phthisis . . . . | 2 |
| Laryngeal phthisis, following pulmonary phthisis . . . . | 7 |
| Cancer of the larynx . . . . . . . . | 1 |
| Syphilis . . . . . . . . . . | 2 |

From all this it appears that: 1. Œdema of the glottis is most commonly due to angina, occurring in the course of some acute or (less frequently) chronic disease. 2. More uncommonly it arises at the termination of chronic laryngitis. 3. In a limited number of cases, simple angina, occurring in healthy persons, may entail an attack of œdema of the glottis.

The invasion of the disease is rarely what can be called absolutely sudden; but it is very often *rapid*—that is, being once developed, it reaches its maximum severity in one, two, or three days.

Pains existed in thirty-two cases in the region of the larynx—pain of great or of trifling severity, or merely constituting a sensation of fulness or of the presence of a foreign body in the part: it was really severe in but five cases. In three persons, fluids, which they attempted to swallow, re-escaped in part through the nose: in one of these the epiglottis was ulcerated; in a second very much infiltrated with serosity; in the third the epiglottis was not diseased at all. The latter case (extracted from M. Louis' work on Phthisis, case xlv,) is the more extraordinary, as nothing existed either in the pharynx or upper part of the glottis to account for the symptom in question.

Cough is only spoken of in fourteen of the cases analysed by M. Valleix. It was frequent in a few instances only; sometimes dry, generally accompanied with expectoration. In one person it was paroxysmal, in another brought on by pressure on the larynx. This symptom appears to have had but slight importance of any kind, except in one instance, where it assumed the *croupal* character.

Change in the character of the voice is among the most invariable symptoms: it is probably even constant. The tone is very rarely croupal, however, having been noted with this peculiarity in but one case,—a different one from that just referred to.

Obstruction of respiration, of greater or less intensity, is the main symptom of the disease: in two cases only was it slight; in all the others, at least from time to time, excessive. But the most remarkable phenomenon connected with respiration in this disease is the inequality of degree of obstruction in inspiration and expiration. M. Valleix has scrutinized the cases in respect of this condition with very particular care; the results are in the following wise. "In twenty-four cases in which the state of the first and second divisions of the respiratory act is carefully noted, *inspiration* is distinctly said to have been whistling or sibilant fifteen times. In eight cases it was noisy, and sometimes attended with peculiar characters; thus M. Legroux discovered a sound like the crow of a cock, the flapping of a valve, a rustling sound, &c.; other persons have found the inspiration hoarse, rattling, &c. In the remaining twenty-fourth case, inspiration is simply said to have been extremely laborious. *Expiration* is only mentioned in eighteen cases, all of them among those in which inspiration was noisy and laborious. In thirteen of them, expiration was free, easy, and unattended with noise, even where inspiration presented in a marked manner the morbid conditions just mentioned. In five persons only did expiration appear laborious; twice it was attended with a noise similar to that accompanying inspiration. It is to be observed further, that in cases where inspiration and expiration were both noted as laborious, the latter was much less so than the former." This difference in the facility with which expiration and inspiration are effected, a character of the disease long known and of great importance in its diagnosis, is however not a pathognomonic sign.

The introduction of the finger has in some instances on record led to the direct discovery of the diseased changes in the form of the prominences caused by the infiltrated structure on each side of the glottis. It is an important fact, as noticed by M. Valleix, that the error of mistaking any other condition for that in question has not been committed by persons employing this procedure.

In all general descriptions of œdema of the glottis great stress, as M. Valleix observes, is laid upon the condition of the face, upon its discoloration, &c. Yet of all the narratives of cases he has analysed, six only contain any distinct announcement of the state of that part. In two of these six cases, the face is said to have continued constantly pale; in one individual livid, in a fourth red, in a fifth anxious, and in the sixth the alæ of the nose were dilated, the face and lips livid and blue. In three cases observed by M. Valleix himself, the face was pale, the lips livid, the features drawn, while the eyes, widely open and hagard, were expressive of anxiety and affright.

Like almost all affections seated in the laynx, œdema of the glottis has a remarkable tendency to paroxysmal course,—this is presumed to depend upon occasional irritation increasing the amount of infiltration, an explanation which cannot be esteemed satisfactory.

Of forty persons attacked with the disease thirty-one died: three only died during a paroxysm; seven in an interval of tranquillity, and under a condition of things where recovery might have been hoped for. In all the other instances death occurred in a state of semi-asphyxia, more or less alarming. One individual perished while tracheotomy was being performed; two others, ten and fifty-two hours after its performance. One person died suddenly.

The disease may last a few hours only, or, even in fatal cases, be prolonged to the twenty-second or twenty-sixth day.

Authors usually insist on the importance of general bleeding in the treatment of the disease. Yet, in some cases only was bloodletting actually had recourse to; and from these no inferences as to its real value can be drawn. The same may be said of the application of leeches. From some cases recorded by the author, it would appear that no doubt can be entertained of the great utility of large blisters and tartarized antimony in emetic doses.

Various surgical means have been employed for the cure of the disease. M. Lisfranc has successfully scarified the prominent rim of tumefied membranes, thus letting out the serosity sufficiently to allow free entry of air. M. Marjolin and M. Legroux have both successfully torn the same parts; the former with a piece of root of marshmallows, the other with the nail cut irregularly into points. M. Valleix next institutes a searching inquiry into the claims of tracheotomy as a curative measure, and concludes that:

"This is without question one of the most valuable modes of treatment which can be employed in œdema of the glottis; recourse should not be had to it until the other means, of which the occasional efficaciousness is established, have been found useless, while, on the other hand, in order to succeed, we should not wait until the patient's strength is exhausted, and until evident signs of asphyxia give evidence of a state of stupefaction of all the organs, from the afflux of non-arterialized blood."

Here, in fact, as elsewhere, the proper moment for operating is to be determined in individual cases by a variety of circumstances.

The practical value of this essay justifies, we trust the reader will admit, the lengthened notice we have bestowed on it.

IV. *Essay on the influence of hereditary transmission in the production of nervous irritability, on the diseases which result therefrom, and the means of curing them,* by M. E. Gintrac. This paper scarcely admits of analysis. Far from being deficient in evidences of talent and power of observation on the part of its author, it is nevertheless principally distinguished by its tiresome verboseness and its numerous grave disquisitions, wherein points utterly problematical are assumed to be settled beyond the reach of cavil. The paper is one redolent of by-gone days, and a system of reasoning which is, fortunately for the solidity of knowledge, fast losing all influence on those who take any active part in advancing the science of medicine.

It may not be without some sort of interest to the reader to have placed before him a list of various affections depending upon "nervous super-excitement:" they are arranged as follows by M. Gintrac, in the presumed order of their tendency to acknowledge hereditary influence. In the first series appear suicidal monomania, mania, lypemania (melancholia), and various nervopathiæ. In a second, convulsions, epilepsy, hysteria, hypochondria, œnomania (a learned term for habitual drunkenness), palpitation, gastralgia, merycism (*id est* rumination, an anomaly of the digestive process which has been observed in a few instances in the human subject, and which M. Gintrac refers to the neuroses of the organ). In a third, chorea, somnambulism, homocidal monomania, theomania, demonomania, choremania, (M. Gintrac does not include under this head the enthusiasts of the Polka and Cellarius Valse, though heaven knows they would to all seeming have a fair claim to be marked as choremaniacs,) neuralgia, asthma, spasm of the larynx, spasm of the œsophagus. In a fourth series appear "acute ataxic states," nervous apoplexy, and trismus of new-born infants. Lastly, pyromania (the mania of setting *our neighbours'* houses on fire, for we do not know that these gentry have any special love for witnessing the conflagration of *their own* property), zoomania, (the mania exhibited by individuals occasionally, from the earliest antiquity, of fancying one's self transformed into an animal,) catalepsy, and tetanus scarcely supply any evidence of hereditary influence.

The following fact is worth being known: A young man, labouring under epilepsy, consulted M. Dumas. The physician, learning that a fit invariably occurred whenever the patient drank spirits, ordered him to take a certain quantity every twelfth day. Having in this way rendered the epilepsy periodical, he cured it with bark.

V. *On the nature and development of adventitious products,* by Dr. C. Baron. This is one of the most painfully tedious productions we have for a length of time been called upon to peruse. The professed object is to show that all adventitious products are nothing more than "transformed blood;" the details are never-ending, the hypotheses startling, the proofs deficient. M. Baron wisely thinks that because people may see cancerous matter and blood mixed together, and because in certain coagula there is an *appearance* of gradual transition from fibrinous into encephaloid substance, that all cancers are nothing more than blood transformed. Such misinterpreted facts might in former days have furnished the respected basis of a popular theory, but in these very material days M. Baron must "charm more wisely," if he desire to rank any of his brethren as disciples of his creed. It has long indeed been felt that John Hunter's notion on the origin of products in the blood does not stand the test of observation; but a complete refutation of it may be found in Dr. Walshe's work (which has just fallen into our hands,) 'On the Nature and Treatment of Cancer.' It may not be out of place to quote the following words from his volume (p. 53), which appear to us most satisfactorily (especially when taken in connexion with the context) to explain the appearances which have deceived M. Baron, and the numerous persons who, *before* him, have held similar views. "It is clear that what has been spoken of by writers as the 'conversion of blood into encephaloid matter,' is nothing more than an appearance produced by the evolution of primary blastema or of absorbed cell-elements in the interstices of a coagulum; the idea of an actual change of blood-corpuscles into cancer-cells is an absurdity."

We confess that the admission of this paper into the Memoirs of the Academy seems to us to bear witness to a fact, already rendered sufficiently clear to the ken of the sharp-sighted by numerous other signs, that the palmy days of French morbid anatomy have passed away.

VI. *On the acute delirium observed in lunatic asylums,* by Dr. Brierre de Boismont. This is a very excellent and well-digested paper; the conclusions to which it leads may be put in the following manner: 1. Acute delirium (phrenitis of the ancients) is neither meningitis nor encephalitis. The etiology of the affection, its morbid anatomy and diagnosis, place this beyond question. 2. It must be regarded as a purely nervous disorder, resembling delirium tremens and traumaticum. Doubtless some modification exists in the cerebral substance, but its nature is as unknown as that existing in the various species of delirium and in various other nervous diseases. 3. The anatomical changes discovered in a certain number of cases are merely accidental or complications of other diseases, or perhaps merely the traces of the alterations of movement. 4. The limits which separate acute delirium from acute mania, from meningitis, and from meningo-cephalitis are not always easily established; indeed it may be said that, under some circumstances, these diseases pass into each other by insensible degrees. These peculiarities and difficulties have betrayed many observers into error, and led them to affirm that acute delirium was identical with meningitis. 5. The causes which produce acute delirium are most closely related to those which engender mental alienation. The influence of moral causes in its generation is likewise very obvious.

6. Acute delirium differs beyond all doubt from mental alienation by its symptomatology, its mode of progress, and its duration; but its similarity to acute mania is in some cases so great, that the observer is tempted to consider it as that affection. 7. Removal from home appears to be advisable, both on account of the dangers to which patients are exposed in their own homes, and on account of the cures which have been effected in asylums. 8. The mode of treatment put in force should vary with the case and the individual. It would be a serious error if, deceived by the febrile state and excitement present, the physician were to employ antiphlogistic measures only. In some cases, where the patient was left entirely to the management of nature, the result has proved favorable.

VII. *Case of extra-uterine interstitial pregnancy*, by Dr. Payan. We agree with the observer and narrator of this case, that although so many as twenty-five narratives of similar description may have been brought together by compilers, still the interest attached to their investigation is by no means exhausted. For this reason we append the chief particulars of the present history, interesting as they are, too, in a medico-legal point of view.

An unmarried woman, aged thirty-two, having already had one child, and again pregnant, occupied as a sort of labourer, was seized one evening after a severe day's work with violent pains in the hypogastrium, accompanied with fainting sensations, thirst, great lassitude, &c. Leeches were applied above the pubis; but the woman expired at two o'clock the following morning. The circumstances of her decease were obviously of a kind to require a judicial investigation. The body was opened in the presence of some of the civil authorities. Measures were taken to expose the parts thoroughly and without rupture.

"The vagina having been laid open at the anterior surface, a few spots of reddish brown colour were seen near the external orifice. There was no sign present of these spots being of recent origin. No trace of blood existed on any part of the mucous membrane of the vagina. The os tincæ and neck of the uterus were sufficiently extensible to allow of the introduction of the little finger. The neck and anterior wall of the uterus having been opened by incision, we discovered in the internal surface of the former a few small patches of reddish-brown colour, without solution of continuity or laceration of any kind. The uterus (which was as large as at about the second or third month of pregnancy) was also cut into at the middle of its anterior surface; the cavity of the uterus was thus laid bare, which was spacious and proportional in size to the development of the uterus; but it did not contain the ovum. It was lined throughout its entire extent, with a concrescible material, of some thickness, a sort of mucous fur, velvety, grayish, forming an imperfectly organized pseudo-membrane, nearly filling the cavity completely. No trace of hemorrhage was at this stage of the proceedings to be seen in the cavity of the uterus. Above this, the proper cavity of the uterus, appeared another cavity, evidently, to my eyes, formed in the substance of the wall of the uterus, and situated towards the upper and superior part of the organ, in the neighbourhood of the uterine extremity of the Fallopian tube. I was unable to assure myself fully whether the latter terminated in this cavity, in consequence of an incision made in the wall of the organ, which rendered it impossible for me to trace the course of the duct."

The cavity of the uterus did not communicate with the cavity in the walls of the uterus containing the fœtus; this seems to us (with the author) unquestionable, though certain persons, apparently with the desire to raise difficulties, maintained that real communication did exist; the uterine tissue forming its wall was so greatly distended, that it had become translucid. Here lay the fœtus uninjured, with the placenta and some almost transparent fluid. The fœtus was of the male sex, about three months old. Death had obviously been caused by accidental rupture of some point of the walls of this cavity, (which was about as large as a duck's egg); a very *large quantity of blood was found within the peritoneum surrounding the uterus.*

This plain explanation of the facts was not accepted without demurrer by some of the medical practitioners present. One of them even started the following mode of accounting for the whole train of circumstances and appearances. This person held that "some foreign body, a gum-elastic bougie, for instance, had been passed into the neck of the uterus during life, between the internal surface of the body of the organ and the membranes, as far as the fundus; that there a perforation had been produced by twisting about the instrument; that next the uterus, thus irritated and excited, had contracted and expelled the ovum through the perforation; and that what I considered to be the fœtal mass in a state of integrity, was nothing more than the ovum rendered adherent to the substance of the uterus by coagula." The criminal attempt which was thus conjectured to have been made to destroy at least the fœtus, obviously had not actually been made at all: the description we have given suffices to show this, and still more satisfactory evidence of the fact may be found in the original.

VIII. *Summary exposition of experiments made upon animals, with the view of ascertaining if the secretion of urine be suppressed in acute and peracute poisoning with arsenious acid.* By Professor O. De Lafond. The experiments of M. Orfila tended to show that the secretion in question does continue under the circumstances mentioned; those of M. Flaudin and Danger would lead to the opposite conclusion. M. de Lafond has attempted to settle the point at issue, and with the following results:

"1. The duration of life, after the administration of the poison in horses, was one hour, one hour and twenty minutes, eight hours, twenty-one, twenty-nine hours, and did not exceed fifty-one hours. In dogs, this duration was five hours, eight hours, and did not exceed twelve hours.

"2. The symptoms observed during life, and morbid changes discovered after death, demonstrated positively the fact of acute or peracute poisoning In some animals, the inflammation of the mucous membrane of the intestine was so violent, as to have excited, in the course of an hour, the formation of several feet in length of canaliculated pseudo-membrane, having the form of the intestine.

"3. In no one of these animals was the urinary secretion suppressed.

"4. The quantity of urine drawn from the bladder with the catheter, expelled naturally, or found in the bladder after death, varied according as the symptoms of poisoning were acute or peracute. The quantity varied in horses between two centilitres and three litres; in dogs, between three centilitres and upwards of a decilitre.

"5. The mean quantity of urine secreted in an hour by animals in good health, compared with that secreted during the same period by those poisoned animals, was, in the case of horses, as 347 : 100; in the instance of dogs, as 6 : 1. Hence, although the secretion is much diminished in quantity, it is not, as advanced by MM. Flaudin and Danger, suppressed.

"6. The urine does not perceptibly contain the poison, until evident symptoms of poisoning show that arsenious acid has been absorbed and circulates with the blood. The time intervening between the administration of the poison and its detection in the urine was not less than one nor more than seven hours.

"7. The arsenical stains, received from the urine by Marsh's tube, on china saucers, were, generally speaking, very brilliant, and presented all the chemical reactions which characterize them in their state of purity."

The experiments, with an abundance of detail (wherefrom the above conclusions are drawn) are related in the original, and may be examined by the sceptical or curious.

IX. *Essay on penetrating wounds of the abdomen, complicated with escape of the omentum,* by Professor Hippolite Larrey. A young man, received a wound (quarter of an inch long) in the abdomen from a knife. In undressing to go to bed, he felt something protrude; a piece of omentum, of the width of two thumbs, had made its way out. No appearance of internal hemorrhage, of gangrene, or serious inflammation taking place, M. Larrey judged it advisable neither to attempt forcible reduction, nor to cut off the part, either by ligature or with the bistoury. The piece of omentum gradually contracted as cicatrization advanced; on the fifty-first day after the receipt of the injury it had totally disappeared, the spot it occupied being covered over by the adhering lips of the wound.

The narrative of the case is followed by a critical examination of the common surgical modes of proceeding in such cases; and the author, in concluding, sagely expresses his hope, that his inquiries upon this question "may help to show, that, in surgery, something else than *cutting, tying, slashing, and in a word destroying,* may be done, and that that something is to temporize opportunely, and, by the simpler resources of art, aid the powerful curative efforts of nature."

---

